# Nitric oxide synthase 2 genetic variation rs2297514 associates with a decreased susceptibility to extremity post-traumatic osteomyelitis in a Chinese Han population

**DOI:** 10.3389/fcimb.2023.1177830

**Published:** 2023-07-03

**Authors:** Chen-sheng Song, Ping Zhang, Qing-rong Lin, Ying-yu Hu, Chun-qiu Pan, Nan Jiang, Yan-jun Hu

**Affiliations:** ^1^ Division of Orthopaedics and Traumatology, Department of Orthopaedics, Nanfang Hospital, Southern Medical University, Guangzhou, China; ^2^ Guangdong Provincial Key Laboratory of Bone and Cartilage Regenerative Medicine, Nanfang Hospital, Southern Medical University, Guangzhou, China; ^3^ Department of Hospital Management, Southern Medical University, Guangzhou, China; ^4^ Department of Emergency Trauma Center, Nanfang Hospital, Southern Medical University, Guangzhou, China

**Keywords:** post-traumatic osteomyelitis, fracture-related infection, Nos2, single nucleotide polymorphisms, rs2297514, case-control study

## Abstract

**Background:**

Previous studies have indicated that nitric oxide synthase 2 (*NOS2*) genetic variations are involved in delayed fracture healing and fracture non-union. Whether these genetic variants associate with the development of osteomyelitis (OM) remains unclear. Here, we analyzed the potential relationships between *NOS2* genetic variations and the risk of developing post-traumatic OM (PTOM) in a Chinese Han population.

**Methods:**

Altogether 704 participants, including 336 PTOM patients and 368 healthy controls, were genotyped of rs2297514 and rs2248814 of the *NOS2* gene using the SNaPshot genotyping method.

**Results:**

Outcomes showed that the frequency of allele C of rs2297514 in the patient group was significantly lower than that in the control group (48.7% *vs*. 54.5%, *P* = 0.029, OR = 0.792, 95% CI 0.642 – 0.976). In addition, significant associations were found between rs2297514 and susceptibility to PTOM by the recessive model (*P* = 0.007, OR = 0.633, 95% CI 0.453 – 0.884), and the homozygous model (*P* = 0.039, OR = 0.648, 95% CI 0.429 – 0.979). Moreover, patients with the CC genotype of rs2297514 had lower inflammatory biomarkers levels than the TT genotype, especially for the C-reactive protein (CRP) level (median: 4.1 mg/L *vs*. 8.9 mg/L, *P* = 0.027). However, no significant relationship was noted between rs2248814 and the risk of developing PTOM.

**Conclusion:**

In this Chinese cohort, rs2297514 is correlated with a decreased risk of PTOM development, with genotype CC as a protective factor.

## 1 Introduction

Osteomyelitis (OM), a hard-to-treat, deep-bone infection, remains a significant healthcare problem worldwide (Muthukrishnan et al., 2019). According to the infection route, OM can occur following perioperative and contiguous conditions, hematogenous spread, and vascular insufficiency-related disorders (e.g., diabetic foot) (Lew and Waldvogel, 1997). Post-traumatic OM (PTOM) remains one primary cause of OM, the incidence of which ranges from 0% to 55%, depending on multiple systematic and local factors of the individuals (Hogan et al., 2013). Despite great advances in surgical techniques, PTOM still poses challenges to orthopaedic surgeons, which primarily attributed to its characteristic of high heterogeneity (Jiang et al., 2020). Early and accurate diagnosis is sometimes difficult, and treatment is always tricky, with high risks of limb deformity and infection recurrence (Panteli and Giannoudis, 2016). Thus, how to reduce the incidence and increase the cure rate is of great clinical significance, which is built on comprehensive understanding of its pathogenesis.

PTOM pathogenesis is complex and associated with both extrinsic and intrinsic factors (Beck-Broichsitter et al., 2015). Most of the previous studies focused on the environmental factors, ignoring the potential role of host factors in developing PTOM. Recently, increasing evidence has suggested that as a representative of host factors, single nucleotide variations (SNVs) were also linked to PTOM development. Such SNVs included but were not limited to rs689466 in cyclooxygenase-2 (COX-2) gene (Wang et al., 2017), rs16944, rs2234663, rs1143627, rs4251961, rs1800796, and rs2234663 in interleukin (IL) genes (Alves De Souza et al., 2017; Jiang et al., 2020). These findings demonstrated that SNVs might play essential roles in PTOM development.

The nitric oxide synthase 2 (NOS2) enzyme, encoded by the *NOS2* gene, is responsible for synthesizing nitric oxide (NO) in the human body. As a reactive free radical, NO mediates multiple biological processes, such as neurotransmission, antitumoral and antimicrobial activities (Huang et al., 2018). In addition, a previous study (Zhu et al., 2001) reported that NOS2 might also participate in the bone fracture healing process, implying that NOS2 plays a role in bone metabolism. Moreover, two recent studies reported *NOS2* SNVs associated with the susceptibility to delayed fracture-healing (Sathyendra et al., 2014) and even fracture non-union (Huang et al., 2018), which confirmed the important role of NOS2 in the fracture healing process.

It is known that PTOM is a bone metabolism-related disorder, characterized by inflammatory bone destruction with or without new bone formation. We speculated that *NOS2* genetic SNVs might also participate in the occurrence of PTOM. Therefore, in the present study, we investigated the potential relationships between *NOS2* genetic SNVs, rs2297514 and rs2248814, and susceptibility to PTOM in a Chinese Han population.

## 2 Materials and methods

### 2.1 Study design, setting, definition, inclusion, and exclusion criteria

The present study was designed as a case-control analysis, with comparison conducted between PTOM patients and healthy controls. Included patients were those who had sought medical attention for PTOM in our hospital between January 2016 and December 2019. Participants in the control group were healthy adults. PTOM is defined as a chronic and persistent inflammatory bone disease by infecting microorganisms, characterized by progressive bone destruction and sequestrum formation following trauma and/or orthopaedic surgery, with infection duration exceeding ten weeks (Metsemakers et al., 2018). PTOM was diagnosed concerning any of the confirmatory criteria outlined by the International Fracture-Related Infection (FRI) Consensus Group (Govaert et al., 2020), including wound breakdown to the bone or the implant, sinus or fistula connecting the bone or the implant, positive pathogen culture outcomes, and positive histopathology test outcomes. Patients with OM following diabetic foot or hematogenous spread, and those who refused to participate were excluded. All the included participants or their legal guardians had signed the informed consent form. This study, conducted following the tenets of the 1964 Helsinki declaration, was approved by the medical ethical committee of Nanfang Hospital, Southern Medical University (NFEC-2019-087).

### 2.2 DNA extraction and SNV genotyping

Peripheral blood samples (5ml each) were collected in the ethylene diamine tetraacetic acid (EDTA) and stored at –80° C. Then, the genomic DNA of each sample was extracted from leukocytes according to the instructions of the Flexi Gene-DNA Kit (Qiagen, Valencia, CA). Two tag SNVs in the *NOS2* gene (rs2297514 and rs2248814) were genotyped using the Multiplex SNaPshot system (Applied Biosystems, Foster City, USA). The forward (F), reverse (R), and extension primers used for polymerase chain reactions (PCR) and extension reactions were as follows: For rs2297514: F: 5’-GCACAGATCAATGAAACCTGC-3’, R: 5’-CGTCTACTCTTGGTTAACCAC-3’, extension primer: 5’-CTGAGAGAGGAAGTGGAGCAGATGCT-3’. For rs2248814: F: 5’-GTCTCCGCTTCTCGTCCT-3’, R: 5’-GGGTGTGAAGGGTCCTCTAC-3’, extension primer: 5’-AGCGGGGTCCTGGCTTGGCTC-3’. The detailed procedure of the SNaPshot genotyping method was described previously (Jiang et al., 2016).

### 2.3 Outcome parameters

Primary outcome measures were comparisons between PTOM patients and healthy controls regarding genotype distribution, mutant allele frequency, and four genetic models (dominant, recessive, homozygous, and heterozygous models) of the two *NOS2* SNVs (rs2297514 and rs2248814). Secondary outcomes were the preoperative serological levels of white blood cell (WBC) count, percentage of polymorphonuclear leukocytes (PMN%), erythrocyte sedimentation rate (ESR), C-reactive protein (CRP), procalcitonin (PCT), interleukin-6 (IL-6), tumor necrosis factor-α (TNF-α), and serum amyloid A (SAA), among different genotypes of the two *NOS2* SNVs. In addition, clinical characteristics of the PTOM cohort were summarized.

### 2.4 Statistical analysis

Statistical analysis was conducted using the Statistical Product and Service Solutions software (version 17.0, SPSS Inc., Chicago, IL, USA). Data distribution was first evaluated for normality by the Kolmogorov-Smirnov test. Continuous variables were presented as mean ± standard deviation (SD) or median with interquartile range (IQR) based on data distribution. For normally distributed data, Student’s t-test or one-way analysis of variance (ANOVA) was used to compare differences between two groups or among three groups. Otherwise, the Mann-Whitney U or Kruskal-Wallis H tests were applied. *Post-hoc* multiple comparisons were conducted using LSD/Dunnett’s T3 following one-way ANOVA or the Mann-Whitney U test following the Kruskal-Wallis H test.

The genotype distributions of the healthy controls were tested to confirm the Hardy-Weinberg Equilibrium (HWE) using the chi-square test. The chi-square test or Fisher’s exact test was used to compare genotype distribution, mutant allele frequency, and the four genetic models, with corresponding odds ratios (ORs) and 95% confidence intervals (CIs) between the patients and healthy controls. All reported values were 2-sided with a *P* value of less than 0.05, which was considered statistically significant.

## 3 Results

### 3.1 Demographics and clinical characteristics

Altogether 468 patients diagnosed with chronic OM (COM) and 368 healthy controls were screened for inclusion, with no statistical differences in sex ratio (0.31 *vs*. 0.37, *P* = 0.25) or median age [48 (IQR 33, 59) years *vs*. 46 (IQR 37, 52) years, *P* = 0.08] between the patients and controls. Of the 468 COM patients, 132 were categorized as non-PTOM (70 having diabetic-foot related OM and 62 having hematogenous spread-related OM), with the remaining 336 patients included for analysis. Among the 336 PTOM patients, traffic accidents accounted for 40% of all injury types, with the tibia (59%) as the most frequent infection site. The positive rate of intraoperative sample culture was 57%, with *Staphylococcus aureus* (46%) being the most frequently detected one.

### 3.2 HWE test outcomes

The *NOS2* genetic variation rs2297514 genotype distribution of the healthy controls failed in the HWE (*P* = 0.04), while the distribution of rs2248814 of the healthy controls was in the HWE (*P* = 0.248).

### 3.3 Potential links between NOS2 gene SNVs and the susceptibility to PTOM

Regarding rs2297514, outcomes revealed a significant difference in genotype distribution between the patients and healthy controls (*P* = 0.026). Further comparison outcome showed that the allele C frequency in the patient group was significantly lower than that in the control group (48.7% *vs*. 54.5%, *P* = 0.029, OR = 0.792, 95% CI 0.642-0.976), demonstrating such a mutant allele may be protective. Additionally, significant links were found between rs2297514 and susceptibility to PTOM by recessive (*P* = 0.007, OR = 0.633, 95% CI 0.453 – 0.884) and homozygous (*P* = 0.039, OR = 0.648, 95% CI 0.429 – 0.979) models (**Table 1**). These suggest that the CC genotype of rs2297514 may be a protective factor against PTOM.

**Table 1:** Relationships between the two *NOS2* genetic variations and susceptibility to PTOM.

SNPs	Items		Patients (n = 336)	Controls (n = 368)	*P* values	OR (95% CI)
rs2297514	Genotype (n, %)	CC	78 (23.2)	119 (32.3)	0.026	
		CT	171 (50.9)	163 (44.3)		
		TT	87 (25.9)	86 (23.4)		
	Allele frequency	C *vs*. T	327/345	401/335	0.029	0.792 (0.642 – 0.976)
	Dominant model	CC+CT *vs*. TT	249/87	282/86	0.437	0.873 (0.619 – 1.230)
	Recessive model	CC *vs*. CT+TT	78/258	119/249	0.007	0.633 (0.453 – 0.884)
	Homozygous model	CC *vs*. TT	78/87	119/86	0.039	0.648 (0.429 – 0.979)
	Heterzygous model	CT *vs*. TT	171/87	163/86	0.846	1.037 (0.718 – 1.497)
rs2248814	Genotype (n, %)	GG	145 (43.2)	172 (46.7)	0.525	
		AG	153 (45.5)	152 (41.3)		
		AA	38 (11.3)	44 (12.0)		
	Allele frequency	G *vs*. A	443/229	496/240	0.559	0.936 (0.750 – 1.168)
	Dominant model	GG+AG *vs*. AA	298/38	324/44	0.789	1.065 (0.671 – 1.690)
	Recessive model	GG *vs*. AG+AA	145/191	172/196	0.340	0.865 (0.642 – 1.165)
	Homozygous model	GG *vs*. AA	145/38	172/44	0.923	0.976 (0.600 – 1.589)
	Heterzygous model	AG *vs*. AA	153/38	152/44	0.539	1.166 (0.715 – 1.900)

SNP, Single nucleotide polymorphism; OR, Odds Ratio; CI, Confidence Interval.

As for rs2248814, no significant relationships were found between this SNP site and the risk of developing PTOM in this Chinese cohort, neither by outcomes of genotype distribution and allele frequency nor by results of the four genetic models (**Table 1**).

### 3.4 Preoperative serological levels of inflammatory biomarkers among different genotypes of the two NOS2 SNV Sites among the PTOM patients

Significant differences were identified regarding the medial levels of ESR (*P* = 0.042) and CRP (*P* < 0.001) among the three genotypes of rs2297514. Outcomes of *post hoc* multiple comparisons by Mann-Whitney U test demonstrated that the medial CRP level of patients with the CC genotype was relatively lower than that of the TT genotype (4.1 mg/L *vs*. 8.9 mg/L, *P* = 0.027). In addition, patients with the CT genotype had significantly lower levels of ESR (*P* = 0.012) and CRP (*P* < 0.001) and a relatively lower IL-6 (*P* = 0.038) level than those of the TT genotype (**Table 2**). Concerning rs2248814, the only positive result was that patients with AG genotype had a relatively higher level of TNF-α than that of the GG genotype (*P* = 0.037) (**Table 2**).

**Table 2:** Preoperative serological levels of inflammatory biomarkers among different genotypes of rs2297514 and rs2248814 in the PTOM patients.

Items	WBC (×10^9^/L)	PMN% (%)	ESR (mm/1h)	CRP (mg/L)	PCT (ng/ml)	IL-6 (pg/ml)	TNF-α (pg/ml)	SAA (mg/L)
rs2297514								
CC	6.9 (5.6, 8.1)	61.7 (51.8, 68.2)	16 (7, 33.5)	4.1 (1.8, 10.3)	0.042 (0.029, 0.057)	6.2 (4.1, 10.5)	8.8 (7.5, 11.9)	11.5 (6.2, 22.7)
CT	6.8 (5.7, 8.1)	59.1 (51.7, 65.8)	13 (6, 29.8)	3.7 (1.2, 7.6)	0.043 (0.030, 0.069)	4.7 (3.2, 11.0)	9.4 (7.6, 12.0)	10.5 (6.2, 18.1)
TT	7.5 (5.8, 9.2)	58.5 (51.2, 65.9)	20 (10, 60.8)	8.9 (3.0, 22.4)	0.046 (0.032, 0.083)	8.1 (3.5, 14.6)	9.3 (7.6, 11.4)	12.1 (5.6, 64.5)
*P* values ^#^	0.295	0.259	**0.042**	**0.000**	0.614	0.085	0.821	0.280
*Post hoc* multiple comparisons ^								
CC *vs*. CT	0.774	0.103	0.311	0.094	0.523	0.234	0.685	0.359
CC *vs*. TT	0.285	0.242	0.223	0.027	0.380	0.258	0.961	0.479
CT *vs*. TT	0.123	0.844	**0.012**	**0.000**	0.534	0.038	0.545	0.138
rs2248814								
AA	7.5 (5.6, 9.3)	59.4 (51.6, 65.4)	20 (10, 48)	5.8 (2.6, 16.0)	0.043 (0.035, 0.074)	5.8 (3.0, 13.2)	9.4 (7.6, 12.2)	11.1 (6.2, 34.6)
AG	6.9 (5.7, 8.3)	59 (51.1, 65.9)	14 (7, 40)	4.1 (1.3, 12.2)	0.041 (0.030, 0.070)	5.9 (3.6, 11.1)	9.5 (7.9, 11.9)	10.2 (6.0, 18.7)
GG	6.8 (5.7, 8.0)	60.2 (51.9, 67.6)	15 (6, 32)	4.6 (1.6, 10.1)	0.045 (0.031, 0.072)	6.1 (3.4, 12.7)	8.3 (7.2, 11.8)	11.6 (6.3, 23.2)
*P* values ^#^	0.472	0.499	0.547	0.228	0.740	0.989	0.104	0.407
*Post hoc* multiple comparisons ^								
AA *vs*. AG	0.276	0.608	0.343	0.097	0.781	0.917	0.936	0.364
AA *vs*. GG	0.228	0.806	0.289	0.250	0.757	0.936	0.232	0.963
AG *vs*. GG	0.850	0.243	0.762	0.381	0.452	0.893	0.037	0.219

PTOM, Post-traumatic osteomyelitis; WBC, white blood cell count; PMN%, percentage of polymorphonuclear; ESR, erythrocyte sedimentation rate; CRP, C-reactive protein; PCT, procalcitonin; IL-6, interleukin-6; TNF-α, tumor necrosis factor-α; SAA, serum amyloid A. ^#^ These P values were obtained by the Kruskal-Wallis H test. ^ These P values were received by the Mann-Whitney U test. Bold values mean statistical significance.

## 4 Discussion

As mentioned previously, successful management of PTOM still represents significant challenges, as early and accurate diagnosis is sometimes difficult. Treatment is often tricky, with infection recurrence risk as high as 20 to 30% (Panteli and Giannoudis, 2016). In addition, such a disorder also brings socioeconomic problems. According to a recent survey of a group of Belgian patients (Iliaens et al., 2021), the direct hospital-related medical care costs of FRI are eight times that of non-FRI of long bone fractures. While in the USA, treatment of bone infection can be up to $150,000 per patient and up to 1.62 billion a year by 2020 (Muthukrishnan et al., 2019). Thus, such heavy economic burdens aggravate the negative influences of PTOM on patients, both physically and psychologically (Walter et al., 2022). Therefore, how to decrease the morbidity and increase the cure rate is clinical significance, built on a comprehensive understanding of PTOM pathogenesis.

Whether PTOM occurs depends on the complex interactions between extrinsic and intrinsic factors, while previous studies primarily focused on extrinsic factors. As a typical representative of intrinsic factors, growing evidence has shown that SNVs also involve in the development of PTOM. Here, we analyzed potential relationships between *NOS2* gene SNVs and susceptibility to PTOM in a Chinese Han population. Outcomes of 704 subjects demonstrated that rs2297514 might be correlated with a reduced risk of PTOM development, with genotype CC as a protective factor. In contrast, the present failed to find a positive link between rs2248814 and the risk of PTOM development in this Chinese cohort. Our findings can be discussed with the following aspects.

First, we found that rs2297514 was associated with a decreased susceptibility to PTOM in this population, with mutant allele C and genotype CC as protective factors. Aside from the current study, two previous studies (Sathyendra et al., 2014; Huang et al., 2018) reported that this SNV was related to the fracture healing process. In 2014, Sathyendra et al. (2014) included 62 participants from the USA and screened 144 SNVs in potentially osteogenic genes. They observed that rs2297514 was linked to an elevated risk of developing atrophic delayed fracture-healing (*P* = 0.015, OR = 3.98), with CT genotype and allele T as risk factors. Later in 2018, Huang et al. (2018) also analyzed this SNV in the development of fracture non-union among a Chinese Han population. Similarly, they also noted that rs2297514 was correlated to an increased susceptibility to fracture non-union, with the T allele as a risk factor. Our present study shared similarities with the two investigations; the mutant allele C as a protective factor in the present study means that the wild-type allele T may be a risk factor for PTOM. However, we found it is the genotype TT, instead of CT, that was identified as a risk factor. Several possible factors might account for the differences, such as different orthopaedic disorders, different ethnicities, and even different numbers of participants.

Second, we failed to find any significant correlations between rs2248814 and the risk of PTOM development in this Chinese cohort, which was in accordance with the study by Huang et al. (2018). However, Sathyendra et al. (2014) reported that such an SNV also increased the risk of delayed fracture healing in an American population. In addition to PTOM, rs2248814 was also reported to be related to several different disorders. Hancock et al. (2008) found that this SNV was a genetic risk factor for Parkinson’s disease. While Velez et al. (2009) observed that rs2248814, interacting with rs1327474, contributed to pulmonary tuberculosis susceptibility in African-Americans. Lim et al. (2013) indicated *NOS2* genetic SNVs (rs2248814 and rs2072324) associated with a sustained virological response to peginterferon plus ribavirin therapy for chronic hepatitis C in Taiwanese Chinese. However, in a recent study (Brookes et al., 2020) focusing on potential relationships between *NOS2* genetic SNVs and susceptibility to the Achilles tendon injuries, the rs2248814 variant might not be linked to the risk of developing Achilles tendinopathy or Achilles tendon rupture. Nonetheless, considering these findings were derived from a single study, more investigations should be conducted to certify these outcomes.

Third, we found PTOM patients with the CC genotype of rs2297514 had relatively lower inflammatory biomarkers (apart from PMN%) levels than those with TT genotype, implying that such an SNV participating in the development of PTOM may partly via its influences on peripheral levels of inflammatory indicators. Interestingly, patients with the CT genotype had significantly lower levels of ESR and CRP and a relatively lower IL-6 than those with the TT genotype, though the heterozygous model found no significant association. Thus, whether the CT genotype is a risk or a protective factor requires further investigations with a larger sample size.

Although we compared serological levels of eight different inflammatory biomarkers among different genotypes of rs2297514 and rs2248814, it is not enough to uncover the underlying mechanisms. It is known that NO, the synthesis of which is regulated by NOS2, is a reactive free radical that plays as an important mediator in neurotransmission, antimicrobial and antitumoral activities (Huang et al., 2018). In addition, previous studies (Zhu et al., 2001; Sathyendra et al., 2014; Huang et al., 2018) had indicated the important role of NOS2 in bone metabolism. Based on these, we speculate that one potential mechanism of *NOS2* SNVs involving in the pathogenesis of PTOM is *NOS2* SNVs may influence expression levels of the NOS2 protein, the latter of which not only affect antimicrobial abilities of the human body, but also affect the bone metabolism process. Of course, future in-depth research is necessary to uncover the detailed mechanisms.

Our study also has several limitations. First, the sample size of the current research remains limited. More eligible patients and controls should be recruited for analysis to obtain more accurate conclusions. Second, the genotype distribution of rs2297514 among the healthy controls was not in HWE; thus, a cautious attitude should be taken, and future studies should testify to such outcomes. Third, we only focused on PTOM; whether such SNVs involve in the occurrence of other OM types, including hematogenous-related OM, and diabetic foot OM, requires further studies. Meanwhile, only two SNVs of the *NOS2* gene were analyzed; whether another SNVs in this gene play a role in the development of PTOM also needs investigation.

## 5 Conclusions

In summary, we found that *NOS2* genetic SNV rs2297514 is associated with a decreased susceptibility to PTOM in this Chinese Han population, with the genotype of CC as a protective factor. Also, such an SNV may play a role partly via its influences on peripheral blood levels of inflammatory biomarkers. Furthermore, the current study failed to find enough evidence to support the hypothesis that rs2248814 is related to PTOM occurrence, which needs to be certified by future studies.

## Data availability statement

The original contributions presented in the study are included in the article/supplementary materials, further inquiries can be directed to the corresponding author/s.

## Ethics statement

The studies involving human participants were reviewed and approved by Southern Medical University Nanfang Hospital. The patients/participants provided their written informed consent to participate in this study.

## Author contributions

C-SS and PZ contributed equally to this study. NJ and Y-JH designed the study. C-SS, Q-RL, Y-YH, and C-QP conducted the experiment. C-SS and NJ performed the statistical analysis. C-SS, PZ, and Q-RL participated in the sample collections. C-SS and PZ drafted the manuscript. NJ and Y-JH contributed to the manuscript revision. All authors contributed to the article approved the submitted version.
